# Supplementary material for: Characteristics influencing COVID-19 testing and vaccination among Spanish-speaking Latine persons in North Carolina
Source: PLoS One. 2025 Jan 23;20(1):e0317794. doi: 10.1371/journal.pone.0317794 (PMC11756757; doi:10.1371/journal.pone.0317794)
Supplement: Attachment — Submitted filename: Response to Reviewers.docx [file pone.0317794.s001.docx]

**PONE-D-24-29547**

**Characteristics Influencing COVID-19 Testing and Vaccination among Spanish-Speaking Latine Persons in North Carolina**

Dear Dr. Weijun Yu,

We are pleased that the academic editor and the reviewers of PLOS ONE found this paper well-written, and we are grateful for the constructive feedback that we received, which has helped to make this paper stronger. We have revised the paper according to the suggestions and have used track changes in sections of the paper where substantial revision has occurred.

We outline below the Journal requirements and the comments received from the reviewers. The changes we have made to the paper in response to each of these comments.

**Journal requirements:**

1. *Please ensure that your manuscript meets PLOS ONE's style requirements, including those for file naming. The PLOS ONE style templates can be found at*

*https://journals.plos.org/plosone/s/file?id=wjVg/PLOSOne_formatting_sample_main_body.pdf and*

[*https://journals.plos.org/plosone/s/file?id=ba62/PLOSOne_formatting_sample_title_authors_affiliations.pdf*](https://journals.plos.org/plosone/s/file?id=ba62/PLOSOne_formatting_sample_title_authors_affiliations.pdf)

**Response:** Thank you for pointing this out. We have reviewed the PLOS ONE style requirements provided in the links above and have updated the manuscript accordingly with the following changes:

- Title was written in sentence case.
- Corresponding author’s initials in parentheses after the email address.
- The level 1 heading for all major sections was changed to 18pt font and in sentence case:
- The level 2 heading for sub-sections was changed to 16pt font:
- The level 3 heading for sub-sections within Level 2 headings was changed to 14pt font and with no italics:
- References: The references are cited in brackets.

2. *We noticed you have some minor occurrence of overlapping text with the following previous publication(s), which needs to be addressed:*

*https://journals.plos.org/plosone/article?id=10.1371%2Fjournal.pone.0296812*

*https://bmjopen.bmj.com/content/12/11/e066585.full*

*In your revision ensure you cite all your sources (including your own works), and quote or rephrase any duplicated text outside the methods section. Further consideration is dependent on these concerns being addressed.*

**Response:** Thank you for bringing this to our attention. The two sources cited are publications produced by our team as part of this project. We have carefully reviewed the manuscript and rephrased some sentences to ensure that no text from the other two publications has been duplicated.

3*. Thank you for stating the following in the Acknowledgments Section of your manuscript:*

“This work is supported by the US National Institute of Minority Health and Health Disparities [U01MD017431; PIs: Scott D. Rhodes and Amanda E. Tanner].”

*We note that you have provided funding information that is currently declared in your Funding Statement. However, funding information should not appear in the Acknowledgments section or other areas of your manuscript. We will only publish funding information present in the Funding Statement section of the online submission form.*

*Please remove any funding-related text from the manuscript and let us know how you would like to update your Funding Statement. Currently, your Funding Statement reads as follows:*

“This work is supported by the US National Institute of Minority Health and Health Disparities [https://www.nimhd.nih.gov/; U01MD017431; PIs: SDR and AET]. The funders had no role in study design, data collection and analysis, decision to publish, or preparation of the manuscript.”

*Please include your amended statements within your cover letter; we will change the online submission form on your behalf.*

**Response:** Thank you for your comment. The funding-related text was removed from the manuscript. We do not want to make any changes to our Funding Statement.

4. *For studies involving third-party data, we encourage authors to share any data specific to their analyses that they can legally distribute. PLOS recognizes, however, that authors may be using third-party data they do not have the rights to share. When third-party data cannot be publicly shared, authors must provide all information necessary for interested researchers to apply to gain access to the data. (*[*https://journals.plos.org/plosone/s/data-availability#loc-acceptable-data-access-restrictions*](https://journals.plos.org/plosone/s/data-availability#loc-acceptable-data-access-restrictions)*)*

*For any third-party data that the authors cannot legally distribute, they should include the following information in their Data Availability Statement upon submission:*

*a) A description of the data set and the third-party source*

*b) If applicable, verification of permission to use the data set*

*c) Confirmation of whether the authors received any special privileges in accessing the data that other researchers would not have*

*d) All necessary contact information others would need to apply to gain access to the data*

**Response.** Thank you for pointing this out. We do not use third-party data. Data are available at <https://radxdatahub.nih.gov/>

5. *Please review your reference list to ensure that it is complete and correct. If you have cited papers that have been retracted, please include the rationale for doing so in the manuscript text, or remove these references and replace them with relevant current references. Any changes to the reference list should be mentioned in the rebuttal letter that accompanies your revised manuscript. If you need to cite a retracted article, indicate the article’s retracted status in the References list and also include a citation and full reference for the retraction notice.*

**Response**: Thank you for your comment. We have carefully reviewed the reference list to ensure that it is complete and correct.

**Reviewers' comments:**

**Reviewer #1:**

- *At the end of the subheading, COVID-19 and Latine Communities in the United States, you mentioned, “Throughout the pandemic and continuing today, these disparities have been notably more pronounced among Spanish speakers and Spanish-speaking immigrants.”*

*Please specify what you mean by Spanish speakers. Are you referring to a first, second or third generation of US-born latines or undocumented latines? Be precise.*

**Response:** Thank you for pointing this out. Our study included Spanish speakers of Latin origin from all immigration statuses, including those born in their country of origin and those born in the United States. We do not have data on whether participants are first, second, or third generation. To simplify our description, we will use the term "Spanish-speaking Latines" and eliminate "Spanish-speaking immigrants."

- *What study you are referring to has been clarified in the paragraph below. Are you referring to the preliminary research you conducted before this study? Please specify that.*

“*Participants in this study reported that many Latines do not trust, are discouraged from using, and face difficulty accessing and navigating the U.S. healthcare system, particularly for non-English speakers and those less familiar with accessing healthcare resources and services in the United States. Participants in this study reported that many Latines do not trust, are discouraged from using, and face difficulty accessing and navigating the U.S. healthcare system, particularly for non-English speakers and those less familiar with accessing healthcare resources and services in the United States.”*

**Response:** We appreciate your bringing this to our attention. This paragraph refers to our community-based participatory research (CBPR) partnership’s preliminary research. We are clarifying and incorporating “preliminary research” instate “study” into the paragraph. We also added a citation for this preliminary research.

- *Methodology section:*

*Study Design:*

1. *Please provide information about the study design used.*

**Response:** We appreciate pointing this out. We added to the Methods section: Cross-sectional baseline data from the parent study are used in this analysis.

*Instrumentation:*

*1. Please describe how the instrument tool used to collect data was organized.*

**Response:** Thank you for your comment. We added to the Measurement section: “The assessment was organized by domain and included sociodemographics, insurance status, immigration documentation, behaviors, testing status and barriers, vaccination status and barriers, and psychosocial factors.”

*2. Did you develop the instrument tool? Please provide this information in the manuscript.*

**Response:** Thank you for pointing this out. We added to Measurement section: Most items were drawn from the RADx-UP Common Data Elements and PhenX toolkit (<https://www.phenxtoolkit.org/>) to ensure standardization of measurement across studies funded by the RADx-UP initiative. Our partnership supplemented these items with additional items that we have used within Latine communities in the US South.

*3. You said that coefficient omega was used to estimate reliability via internal consistency for social support. What about the other items in the instrument tool? Did you estimate their reliability as well?*

**Response:** Thank you for your comment. Social support has 3 items; it is the only scale in this analysis for which omega use is appropriate.

*4. Did you assess the validity of the instrument tool?*

**Response:** Thank you for your question. Yes, as we noted, “All measures have been validated among Spanish-speaking Latine populations living in the United States previously.”

*5. If the instrument has been developed by someone else, please specify the type of validity and reliability conducted by the authors who created the instrument tool.*

**Response:** Thank you for your comment. We have detailed in the Dependent variables and Independent variables sections where items on the assessment originated.

*6. If you did not perform the validity and reliability of the instrument tool, please include these in the limitation section of your study.*

**Response:** Thank you for your comment. We have detailed in the Dependent variables and Independent variables sections where items on the assessment originated.

- Participants

*You said that you used 20 “navegantes” and their social networks to recruit participants.*

1. *How many times did each “navegante” have to contact potential participants?*

**Response:** Thank you for pointing this out. Regarding recruitment, we do not have a record of how many times each Navegante contacted potential social network participants. The Navegantes provided us with their lists of eight social network participants only after social network participants had agreed to participate.

*2. Did you follow a specific methodology for contacting participants, such as the modified tailored design method (Dillan, 2014)? Please describe the methodology followed.*

**Response:** We appreciate this important question. As we describe in our Study Setting and Participants section, because each *Navegante* must be a leader within existing social networks, we primarily recruited *Navegantes* through our partnership’s extensive community networks across North Carolina and through word-of-mouth. Each *Navegante* was responsible for recruiting eight participants from their social network; they generally did this through in-person communication.

- *Independent Variables:*

*You said: “Eight barriers to testing and 10 barriers to vaccination were assessed, including taking time off from work, out-of-pocket costs, not knowing where to go, pain or discomfort, and worries about confidentiality. Please name all the barriers to testing and vaccination.*

**Response:** Thank you for pointing this out. Below we list the barriers according to our baseline.

Barriers to getting tested:

1. Need to take time off work to get tested
2. Out of pocket costs for test
3. Out of pocket costs for transportation, childcare, or time off work
4. I do not know where to go to be tested
5. Pain or discomfort from the test
6. Saliva
7. Concern about others handling my personal data
8. Other

Barriers to getting vaccinated:

1. I’m allergic to vaccines

2. I don’t like needles

3. I’m not concerned about getting really sick form COVID-19

4. I’m concerned about side effects from the vaccine

5. I don’t think vaccines work very well

6. I don’t trust that the vaccine will be safe

7. I don’t believe the COVID-19 pandemic is as bas as some people say it is

8. I don’t want to pay for it

9. I don’t know enough about how well a COVID-19 vaccine works

10. Other

We also have included them in the text of the paper.

- Data Analysis:

1. *How did you analyze the data? Did you use a software such as SPSS, JMP, R, or Excel? If so, please describe the software utilized for data analysis and the version.*

**Response:** Thank you for your question. All analyses were performed in SAS v9.4 (SAS Institute, Cary, NC). We are adding this statement to the paper in the data analysis section.

2. *For those unfamiliar with the Akaike information criterion, please briefly describe what it is used for, explain the best-fit model you found, and state the AIC weight of the model.*

**Response**: Thank you for your comment. We are adding to the manuscript the following statement: “AIC is useful for comparing models in a relative sense between two or more candidate regression models. The AIC calculation is a function of the number of independent variables used to build the model and the estimate of the likelihood for the model. The lower the AIC score the better.”

- **Discussion**

*The author engages in three steps in a discussion section: interpretation, analysis, and explanation. An adequate discussion section will also explain why the research results are essential and where they fit in the current literature while being self-critical and honest about the study's shortcomings. In your discussion, you must mention how your data fits the literature. The results from your research are similar/different to those found by other authors? Also, you must make more explicit about the novelty that your study found and what your contributions to the literature are.*

**Response:** Thank you for your insightful feedback on the discussion section of our manuscript. We have made revisions to clarify our findings and their significance with existing literature. You can find a revised version of the discussion in the paper that incorporates your suggestions.

**Reviewer # 2:**

Thank you for the opportunity to review this paper. Overall, this is a well-written paper. I have few comments detailed below.

- *Overall: The title and most of the paper indicates that the research was conducted among immigrant Latine population, however, upon reviewing this paper in entirety, it does not seem to be true. The authors have included all Latine population of the specified geographic region, irrespective of their immigration status. The authors should consider clarifying this throughout the manuscript.*

**Response:** Thank you for highlighting this important point. Our study included Spanish speakers of Latino origin from various immigration statuses, although the majority were born in their home countries (89.9%), and later immigrated to the United States. To clarify, we will consistently use the term "Spanish speakers" throughout the manuscript, and in some sections, we specify that they are predominantly immigrants.

- *Abstract: The authors mention challenges to COVID testing/vaccinations in their conclusions but do not report the challenges in the results. The authors should consider updating the results with the said challenges.*

**Response:** Thank you for pointing this out. We are updating the results and conclusion in the Abstract to enhance clarity.

- *Introduction: While the authors focus their research question on Latine immigrants, they report the COVID vaccinations among overall Latine population and is not specific to Latine immigrants. The authors should consider clarifying the population and update the introduction to report existing literature and the research gap based on that*.

**Response:** Thank you for highlighting this point. Our study included Spanish speakers of Latino origin from various immigration statuses, although the majority were born in their home countries (89.9%), and later immigrated to the United States. To enhance clarity, we will use the term "Spanish speakers Latines".

- *Methods: It seems like authors have assessed all Latinas in NC and not just immigrants. This information is confusing based on the title and abstract that focuses on immigrants only. The authors should consider clarifying if overall Latine population of NC constituted the study cohort or just Latine immigrant population.*

**Response:** Thank you for emphasizing again this point. We confirm that our study included Spanish speakers of Latino origin from various immigration statuses, although the majority were born in their home countries (89.9%), and later immigrated to the United States. To enhance clarity, we are using just the term "Spanish-speaking Latine" in this section.

- *Discussion: The discussion currently is only an extension of the results where the authors explained and interpreted the findings. The authors should consider updating the discussion with comparison of the findings related to COVID testing/vaccination, challenge, barriers etc, with other races (non-Hispanic White/ Black) in NC/United States. Else, it is hard to understand whether the testing/vaccinations rates, or challenges are indeed lower/higher/comparable to the other races, and if any interventions are required for this specific population.*

**Response:** Thank you for your insightful comment. We agree that conducting a comparative analysis would be very interesting. However, this study was specifically designed and approved to evaluate the behaviors and acceptance of COVID-19 resources among a specific population: Spanish-speaking Latines, rather than comparing them with other racial groups. In this study, we do not have the data necessary to conduct a comparative analysis of different racial groups within the same location and timeframe. We believe your suggestion is excellent and could serve as a valuable avenue for future research.

- *The authors should exercise caution with conclusions especially since the sample size is very small.*

**Response**: We appreciate you bringing this to my attention. We have updated our conclusions to make them more precise.

- *One of the limitations states that the findings cannot be generalized to the overall Latine population, would the authors clarify the study cohort throughout this manuscript, whether they are immigrant Latine population or overall Latine population of NC.*

**Response:** Thank you for your comment in this section. We confirm that our study included Spanish speakers of Latino origin from various immigration statuses living in North Carolina. To be more specific we are using the term " Spanish-speaking and immigrant Latines in North Carolina" in this section.

- *The authors should consider adding strengths of this study*

**Response:** We appreciate your comment and suggestion. We are adding a strength section to this study.

We would like to express again our gratitude to the academic editor and reviewers of PLOS ONE for their valuable comments. We have carefully considered each piece of feedback and believe we have addressed all of them. We hope our responses meet your expectations, and we look forward to continuing the process of publishing this relevant article.

Sincerely,

Sandy K. Aguilar-Palma

Senior Research Associate

Department of Social Sciences and Health Policy

Division of Public Health Sciences

Wake Forest University School of Medicine

E-mail: [saguilar@wakehealth.edu](mailto:saguilar@wakehealth.edu)
